# A National Advisory Committee on Immunization (NACI) update on invasive meningococcal disease (IMD) epidemiology and program-relevant considerations for preventing IMD in individuals at high risk of exposure

**DOI:** 10.14745/ccdr.v49i09a01

**Published:** 2023-09-01

**Authors:** Anne Pham-Huy, Joseline Zafack, Courtney Primeau, Oliver Baclic, Marina Salvadori, Shelley Deeks

**Affiliations:** 1 Department of Pediatrics, Children’s Hospital of Eastern Ontario, University of Ottawa, Ottawa, ON; 2 Centre for Immunization Programs, Public Health Agency of Canada, Ottawa, ON; 3 Centre for Immunization and Respiratory Infectious Diseases, Public Health Agency of Canada, Ottawa, ON; 4 Department of Pediatrics, McGill University, Montréal, QC; 5 Nova Scotia Department of Health and Wellness, Halifax, NS

**Keywords:** National Advisory Committee on Immunization, NACI, invasive meningococcal disease, adolescents and young adults, meningococcal vaccine, vaccination policy, guidance

## Abstract

Following recent outbreaks of invasive meningococcal disease (IMD) in Canada and updates to provincial vaccination guidelines, the National Advisory Committee on Immunization (NACI) conducted a targeted review of evidence with a focus on immunization of adolescents and young adults. NACI reviewed national and international immunization recommendations for populations at high-risk of IMD, national IMD epidemiology and program-relevant considerations. Given the varied IMD epidemiology, NACI determined that recommending a pan-Canadian targeted program is currently challenging and that regional programs may be better suited to prevent IMD in population groups considered to be at high-risk of exposure. Further data is needed to ascertain contemporary risk factors for IMD (including activities and settings associated with bacterial acquisition, carriage and transmission) and estimate the true cost of meningococcal vaccine-preventable infections in Canada. To support provinces and territories in their decision-making, an outline of program-relevant elements for provincial and territorial consideration is provided.

## Introduction

Invasive meningococcal disease (IMD) is a rare but serious bacterial disease with a relatively high case fatality rate and significant long-term sequelae, including limb amputations and permanent central nervous system injury ((1)). Following the recent cases of IMD on university campuses in the winter of 2022/2023 in Atlantic Canada ((2)) as well as the subsequent recommendations for the immunization of post-secondary students and other young adults living in congregate living settings by some provinces and territories (PTs) ((3,4)), the Public Health Agency of Canada and the National Advisory Committee on Immunization (NACI) were requested by the Council of Chief Medical Officers of Health to review the current national guidance on use of serogroup B meningococcal vaccines and quadrivalent conjugate meningococcal (Men-C-ACYW) vaccine boosters in post-secondary settings. Specifically, the policy question reviewed by NACI was, “Should additional high-risk populations be offered a serogroup B meningococcal vaccine and/or Men-C-ACYW booster vaccine in order to prevent IMD outbreaks in older adolescents and young adults, 15 to 24 years of age?”

## Methods

To answer the policy question, NACI conducted a targeted review of evidence, with a focus in adolescents and young adults, that included national and international guidelines for the prevention of IMD in populations at high risk of IMD exposure, national and international definitions of high-risk populations, meningococcal vaccine characteristics, and EEFA (ethics, equity, feasibility, acceptability) programmatic considerations related to immunization of individuals at high risk of IMD exposure. The epidemiological data for IMD cases with disease onset between January 1, 2012 and December 31, 2019, in Canada was obtained from a previous analysis ((5)). The Public Health Agency of Canada compiled updated Canadian epidemiological data, including an outbreak analysis for IMD cases occurring between 2020 and 2022, through a data request to PTs participating in the National Enhanced Invasive Meningococcal Disease Surveillance System, through which PTs voluntarily report epidemiologic data on confirmed IMD cases on an annual basis. A request was made to PTs not currently participating in the National Enhanced Invasive Meningococcal Disease Surveillance System to also obtain these data for the same period. The data were validated for 12 of the 13 PTs, while the analyses for the remaining PTs was based on the isolate submissions provided to the National Microbiology Laboratory for confirmation of serogroup and further strain characterization. All age-standardized analyses were done with the direct method using the 2011 Canadian census data. The NACI IMD Working Group met on May 10 and 24, 2023, and the full committee reviewed the evidence presented to the NACI IMD Working Group on June 5, 2023. NACI approved the conclusions on July 14, 2023.

## National and international immunization recommendations

Currently, NACI recommends that adolescents and young adults, depending on local epidemiology and programmatic considerations, receive a dose of monovalent conjugate meningococcal C (Men-C-C) or quadrivalent Men-C-ACYW vaccine routinely at the age of 12 (grade six or seven), even if previously vaccinated as infants or toddlers ((6)). NACI also recommends the use of protein-based meningococcal vaccines that primarily target serogroup B (serogroup B meningococcal vaccines: Bexsero™, 4CMenB; or Trumenba™, MenB-fHBP) on an individual basis, taking into consideration the individual preferences, regional serogroup B epidemiology and strain susceptibility. For individuals at high risk of IMD due to exposure or underlying medical conditions, NACI recommends immunization with a serogroup B meningococcal vaccine and Men-C-ACYW vaccine, as well as Men-C-ACYW booster immunization for those at ongoing risk ((7)).

In Canada, the adolescent dose of Men-C-ACYW is primarily provided through school-based immunization programs in grades four through 12 (children 9–17 years of age) ((8,9)). Eight PTs (Prince Edward Island [PE], British Columbia [BC], Alberta [AB], Nunavut [NU], New Brunswick [NB], Yukon [YT], Northwest Territories [NT], Québec [QC]) currently provide vaccination in grade nine or later, typically less than five years prior to the initiation of post-secondary studies. Based on a generally accepted assumption that protection from vaccination lasts at least five years, immunization offered through late adolescent school-based programs is likely to see protection last into the first years of post-secondary settings. Recently, PE and Nova Scotia [NS] also expanded their IMD programs to include serogroup B meningococcal immunization of adolescents and young adults who are living in group settings while attending post-secondary education (e.g. living in dormitory or other residence) and living for the first time in a youth-based congregate living setting, respectively ((3,4)).

In 2021, 89% of 17-year-old adolescents in Canada had received at least one dose of meningococcal vaccine, which is consistent with the national goal of 90% vaccine coverage at this age ((10,11)). In addition, through its current immunization programs, Canada has also been able to achieve its disease reduction goal of fewer than five cases per year of IMD caused by serogroup C in children younger than 18 years of age ((10)). Most cases of serogroup C IMD currently occur in unvaccinated adults over 40 years of age ((5)).

While the majority of IMD cases in Canada are sporadic, outbreaks have occurred across the country with variable magnitudes. As part of a comprehensive public health responses to these outbreaks, Canadian PTs have previously implemented targeted immunization programs ((12)). Most recently, meningococcal vaccines have been used to control hypervirulent serogroup B (ST-269) and W (ST-11) clones at the provincial or regional level in QC, BC and AB ((12,13)).

Internationally, several jurisdictions recommend catch-up or an additional dose of meningococcal vaccine to adolescents and young adults who are attending post-secondary studies or living in close quarters, including university students living in residential colleges and residential accommodation. The United States, United Kingdom, Australia and New Zealand identify post-secondary students, particularly those during the first year of attendance and those residing in close-living situations, as being at increased risk of IMD and have recommended vaccination ((14–17)). Increased relative risk for serogroup B IMD in these jurisdictions has previously been estimated to be approximately three times higher for students compared to non-students in the same age group ((18,19)).

### Epidemiology of invasive meningococcal disease in Canada, 2012–2022

NACI reviewed the epidemiological risks associated with different serogroups of IMD in Canada by age group and geography. Since the introduction of meningococcal immunization programs in the early 2000s, the epidemiology of IMD in Canada has changed significantly. The incidence of IMD due to serogroup C declined by 93% and the overall IMD incidence declined by 55% from the pre-vaccine era to 2015 ((20)).

**Figure 1:**
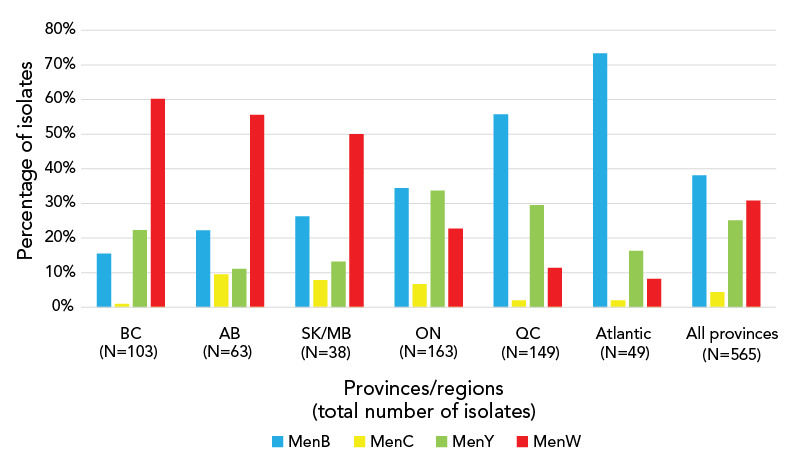
Serogroup distribution of invasive meningococcal disease case isolates^a^ by province/regions, 2015–2020^b^ Abbreviations: AB, Alberta; BC, British Columbia; MB, Manitoba; MenB, *Neisseria meningitidis* serogroup B; MenC, *Neisseria meningitidis* serogroup C; MenW, *Neisseria meningitidis* serogroup W; MenY, *Neisseria meningitidis* serogroup Y; ON, Ontario; QC, Québec; SK, Saskatchewan ^a^ Total includes other serogroups (e.g. E, Z, non-encapsulated) ^b^ Atlantic includes New Brunswick, Nova Scotia, Prince Edward Island, Newfoundland and Labrador Data source: National Microbiology Laboratory

Between 2012 and 2022, there were a total of 1,196 cases of IMD reported in Canada. Overall, the mean incidence of IMD during this period was 0.31 cases per 100,000 population per year (**Table 1**); however, the distribution according to the number of cases, incidence rates and serogroups varied substantially across age groups and PTs (**Figure 1**).

**Table 1:** Incidence rates, per 100,000 population, of invasive meningococcal disease in Canada by age group and year, 2012–2022 (N=1,178 cases^a^)

**Age group** **(years)**	**2012**	**2013**	**2014**	**2015**	**2016**	**2017**	**2018**	**2019**	**2020^b^**	**2021^b^**	**2022^b^**	**2012–2022** **(mean)**
**Younger than 1**	3.42	3.65	4.96	2.86	1.56	3.38	4.20	4.46	2.16	1.94	1.63	3.11
**1–4**	1.18	1.17	0.97	0.91	0.84	0.51	0.83	1.02	0.65	0.39	0.60	0.82
**5–9**	0.44	0.27	0.10	0.05	0.20	0.20	0.05	0.15	0.20	0.10	0.15	0.17
**10–14**	0.68	0.16	0.11	0.21	0.10	0.16	0.05	0.10	0.00	0.10	0.28	0.18
**15–19**	1.17	0.73	0.84	0.66	0.52	0.71	0.47	0.47	0.14	0.19	0.47	0.58
**20–24**	0.51	0.25	0.17	0.33	0.25	0.79	0.37	0.53	0.36	0.24	0.28	0.37
**25–29**	0.25	0.25	0.12	0.12	0.16	0.08	0.12	0.39	0.15	0.11	0.18	0.18
**30–39**	0.13	0.13	0.06	0.15	0.10	0.10	0.16	0.20	0.19	0.07	0.09	0.13
**40–59**	0.31	0.16	0.18	0.20	0.13	0.15	0.31	0.20	0.19	0.09	0.15	0.19
**60 and older**	0.28	0.42	0.22	0.33	0.41	0.42	0.52	0.42	0.20	0.06	0.14	0.31
**Overall (crude rate)**	0.45	0.35	0.29	0.30	0.27	0.33	0.37	0.37	0.23	0.13	0.21	0.30

^a^ N=18 cases of total sample were missing age

^b^ Coronavirus disease 2019 (COVID-19) pandemic, public health measures (e.g. lockdown, physical distancing) were implemented

Data sources: National Enhanced Invasive Meningococcal Disease Surveillance System (eIMDSS), data request to provinces and territories not participating in eIMDSS, and National Microbiology Laboratory

When considering age, the highest annual incidence rates between 2012 and 2022 were observed for infants younger than one year of age (mean incidence: 3.11 cases per 100,000 population), followed by children 1–4 years of age (0.82 cases per 100,000 population). Adolescents 15–19 years of age and young adults 20–24 years of age had slightly lower mean incidence compared to children 1–4 years of age at 0.58 cases per 100,000 population and 0.37 cases per 100,000 population, respectively. From 2012 to 2022, children younger than five years of age accounted for the largest number of cases (N=265, or 23% of total IMD cases) followed by adolescents 15–19 years of age (N=138, or 12% of total IMD cases) and adults 20–24 years of age (N=98, or 8% of total IMD cases).

Between 2012 and 2022, the highest incidence of IMD was serogroup B (0.14 cases per 100,000 population), followed by serogroup W and serogroup Y (both 0.06 cases per 100,000 population, respectively). Serogroup B incidence was highest in children younger than one year of age and children in the 1–4 years age group (2.03 and 0.59 cases per 100,000 population, respectively), followed by serogroup W in children younger than one year of age (0.48 cases per 100,000 population) and serogroup B in the 15–19 and 20–24 years age groups (0.34 and 0.17 cases per 100,000 population, **Table 2**). During this period, the highest number of cases were reported for serogroup B in the younger than five (N=189), 15–19 (N=79) and 20–24 (N=46) years age groups. This was followed by cases caused by serogroups W and Y in children younger than five years of age (N=39 serogroup W cases), adolescents 15–19 years of age (N=15 and N=30 serogroup W and Y cases, respectively) and young adults 20–24 years of age (N=18 and N=23 serogroup W and Y cases, respectively).

**Table 2:** Incidence rates, per 100,000 population, of invasive meningococcal disease in Canada by age group and serogroup, 2012–2022 (N=1,178 cases^a^)

Serogroup	Age group (years)										Overall
	Younger than 1	1–4	5–9	10–14	15–19	20–24	25–29	30–39	40–59	60 and older	
B	2.03	0.59	0.12	0.10	0.34	0.17	0.10	0.05	0.06	0.09	0.14
C	0.07	0.01	0.01	0.01	0.02	0.02	0.00	0.02	0.02	0.02	0.02
W	0.48	0.09	0.01	0.01	0.06	0.06	0.04	0.02	0.05	0.08	0.06
Y	0.17	0.02	0.01	0.03	0.13	0.07	0.03	0.02	0.05	0.09	0.06
Non-groupable	0.00	0.00	0.00	0.00	0.00	0.00	0.00	0.00	0.00	0.00	0.001
Other^b^	0.00	0.00	0.01	0.00	0.01	0.01	0.00	0.00	0.00	0.00	0.003
Unknown^c^	0.24	0.06	0.01	0.01	0.03	0.01	0.01	0.01	0.01	0.01	0.02

^a^ N=18 cases of total sample missing age

^b^ Other are those cases with the serogroup noted as: A, E, Z, 29E and non-encapsulated

^c^ Unknown are those cases missing serogroup information

Data sources: National Enhanced Invasive Meningococcal Disease Surveillance System (eIMDSS), data request to provinces and territories not participating in eIMDSS, and National Microbiology Laboratory

Most PTs had an annual mean IMD incidence rate of less than 0.50 cases per 100,000 population (**Table 3**). However, the age-standardized incidence rates were highest in NU (1.15 cases per 100,000 population per year, 95% CI: 0.28–2.00), followed by NT (0.49 cases per 100,000 population per year, 95% CI: 0.07–1.10), NS (0.47 cases per 100,000 population per year, 95% CI: 0.27–0.66) and QC (0.44 cases per 100,000 population, 95% CI: 0.29–0.59).

**Table 3:** Age-standardized incidence rates^a^, per 100,000 population, of invasive meningococcal disease by province/territory and year

Provinces and territories	2012	2013	2014	2015	2016	2017	2018	2019	2020	2021	2022	2012–2022 Mean (95% CI)
British Columbia	0.36	0.24	0.29	0.22	0.18	0.54	0.52	0.52	0.25	0.13	0.08	0.30 (0.19–0.41)
Alberta	0.41	0.35	0.22	0.28	0.19	0.20	0.58	0.32	0.28	0.19	0.10	0.28 (0.20–0.37)
Saskatchewan	0.26	0.24	0.24	0.07	0.16	0.26	0.21	0.17	0.23	0.1	0.27	0.20 (0.15–0.25)
Manitoba	0.16	0.66	0.23	0.36	0.60	0.41	0.37	0.48	0.27	0.34	0.53	0.40 (0.30–0.51)
Ontario	0.26	0.17	0.19	0.25	0.20	0.22	0.26	0.25	0.16	0.07	0.17	0.20 (0.16–0.24)
Québec	0.92	0.74	0.47	0.43	0.40	0.41	0.29	0.44	0.30	0.14	0.28	0.44 (0.29–0.59)
New Brunswick	0.83	0.45	0.26	0.72	0	0.16	0.95	0.58	0	0.31	0.08	0.39 (0.17–0.62)
Nova Scotia	0.10	0	0.35	0.82	0.44	0.66	0.70	0.60	0.34	0.26	0.85	0.47 (0.27–0.66)
Newfoundland and Labrador	0.24	0	0.41	0	0.68	0.62	0	0.71	0.45	0.23	0.41	0.34 (0.16–0.52)
Prince Edward Island	0.76	0	0	0.70	0	0	0	0	0	0	0	0.13 (0.07–0.33)
Yukon	0	0	0	0	0	0	2.30	0	0	0	0	0.21 (0.03–0.67)
Northwest Territories	1.79	0	0	0	1.87	0	0	1.76	0	0	0	0.49 (0.07–1.10)
Nunavut	1.34	0	3.74	0	0	1.28	2.51	2.36	0	1.38	0	1.15 (0.28–2.00)

^a^ Direct method age standardization using the 2011 Canadian census data

Data sources: National Enhanced Invasive Meningococcal Disease Surveillance System (eIMDSS), data request to provinces and territories not participating in eIMDSS, National Microbiology Laboratory

Recently, trends in geographical differences across Canadian jurisdictions have been observed for prevalent serogroups. Between 2015–2020, culture-confirmed IMD due to serogroup W was common in Western Canada, accounting for more cases (60.2% and 55.6% of IMD cases in BC and AB, respectively) than all other serogroups combined. In contrast, IMD due to serogroup B was more common Eastern Canada in QC and Atlantic Canada, accounting for 55.3% and 73.3% of IMD cases, respectively (Figure 1).

**Figure 2:**
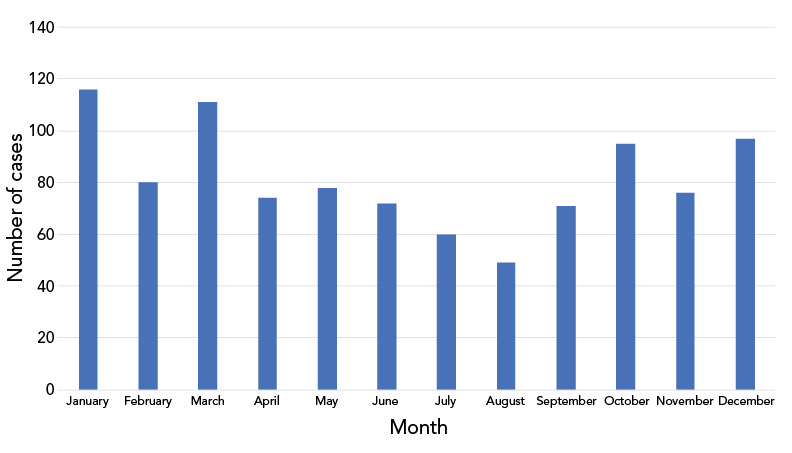
Number of invasive meningococcal disease cases in Canada by month, 2012–2022 (N=979^a^) ^a^ Month of onset was unknown for 199 cases Data sources: National Enhanced Invasive Meningococcal Disease Surveillance System (eIMDSS), data request to provinces and territories not participating in eIMDSS, and National Microbiology Laboratory

Overall, the majority of IMD cases in Canada occurred in the fall and winter months with the peak onset observed in the month of January (N=116, 11.8%), March (N=111, 11.3%) and December (N=97, 9.9%) (**Figure 2**).

Based on the genetic testing of IMD strains from Canadian provinces that was conducted by the National Microbiology Laboratory for the period from 2010 to 2020, over 90% of serogroup B strains have been predicted to have a sufficient antigen expression that would elicit an immune response in individuals vaccinated with either of the two currently authorised serogroup B vaccines ((21–23)). Similarly, based on the level of bacterial antigen surface expression determined by the meningococcal antigen typing system, 4CMenB vaccine was previously predicted to confer protection against a high proportion of serogroup B IMD isolates collected between 2010 and 2014 from all parts of Canada ((21)).

### Vaccine protection against invasive meningococcal disease

High levels of antibody are important for protection against IMD due to the rapid disease progression and because bactericidal activity (the presumed primary immunologic mechanism of protection) is predominantly achieved through antibody-mediated complement activation ((24,25)). Available data suggest that protection against IMD decreases in many adolescents and young adults within five years following immunization with conjugate meningococcal vaccines ((14,17,26–28)). While vaccine effectiveness data are limited for serogroup B vaccines, it is likely that over 50% of vaccine recipients maintain protection up to four years post immunization ((13,29–34)). Serogroup B vaccines are also likely to broaden the protection against non-B serogroups expressing the vaccine-contained antigens. While not authorized for this indication, 4CMenB may also provide some cross-protection against *Neisseria gonorrhoeae* ((29,35–37)). However, neither of the serogroup B vaccines appear to have an effect on carriage and, consequently, herd immunity ((38–40)).

### Invasive meningococcal disease risk assessment and program-relevant considerations

In addition to the assessment of the national disease burden, vaccine characteristics and existing PT immunization programs, NACI also considered EEFA program-relevant elements in the context of the Canadian Immunization Guide definitions and NACI recommendations for high-risk groups due to increased risk of exposure.

Based on the principle of equity and ethics, it was acknowledged that all population groups identified as being at high risk of exposure should be equally considered for, and have access to, vaccination against IMD. However, it was also recognized that, given the very small number of cases, as well as due to high cost of meningococcal vaccines, immunization of all individuals who may be at increased risk of exposure may not be equally feasible across PTs.

In general, NACI concluded that, given the diversity of the Canadian health care system and differences in the individual PT disease burden, it was important to allow flexibility for PTs to make individual decisions about which populations they wish to prioritize for immunization. However, while the level of risk is influenced by local epidemiology and the timing and type of adolescent PT programs, it was recognised that any permissive recommendations should not lead to increased inequity relative to vaccine access (e.g. depending on the place of residence or ability of high-risk individuals to purchase the recommended vaccines).

To support PTs in their decision making, NACI provided an outline of program-relevant elements to be considered when assessing the population-group risk and deciding on whether an IMD program for that population is warranted (**Table 4**).

**Table 4:** Program-relevant considerations when deciding on the introduction of immunization programs for groups at high risk of invasive meningococcal disease exposure

Program-relevant factors	Elements for consideration
Epidemiology and risk factors	· IMD is a rare (approximately 100 cases per year over the last decade) but serious disease with high case fatality and life-long sequelae. · IMD epidemiology varies between Canadian PTs, with IMD incidence being highest in individuals younger than five years of age and followed by those in the 15–24-year-old age group. · While there were regional differences in serogroups causing IMD in 2012–2022, serogroup B disease represents the largest proportion of IMD cases, including in individuals 15–24 years of age. · Population activities and settings that have previously been associated with increased risk of IMD may have changed over the last several decades, and no activities or settings were identified through the available Canadian epidemiological data as leading to increased risk of IMD in Canada. Age remains a highly reliable predictor of risk.
Vaccine characteristics	· High levels of antibody are important for protection against IMD due to the rapid disease progression and because bacterial killing is primarily achieved through antibody-mediated complement activation ((24,25)). · Available data suggests that protection against IMD decreases in many adolescents and young adults within five years following immunization with conjugate meningococcal vaccines ((14,17,26–28)) or serogroup B vaccines. · Based on the genetic testing, over 90% of recent serogroup B isolates in Canada have been reported to express antigens at levels that are predicted to be susceptible to the bactericidal immune response elicited following the vaccination with serogroup B vaccines ((21–23)). · Although it is currently not authorized for protection against *N. gonorrhoeae,* outer membrane vesicles-based vaccines, such as the 4CMenB vaccine, have been reported in small studies to potentially offer some level of cross-protection against gonococcal infection ((29,35–37)). Clinical trials evaluating vaccine effectiveness are ongoing (NCT04350138). · Both serogroup B and serogroup C-containing vaccines authorized for use in Canada have an acceptable safety profile ((30,41)). · Booster vaccination with Men-C-ACWY vaccine five years following the primary schedule is safe and recommended by NACI for preventing IMD in high-risk individuals.
Ethics and equity	· Equity deliberations should take into consideration the variation in IMD burden in different population groups with the goal of reducing inequity in disease outcomes. · When considering age, the highest annual incidence rates between 2012 and 2022 were generally observed for infants less than one year of age, followed by children 1–4 years of age. Adolescents 15–19 years of age and young adults 20–24 years of age had slightly lower mean incidence compared to children 1–4 years of age. · Previously conducted studies have shown high rates of *N. meningitidis* acquisition and carriage in late adolescence and young adulthood ((18,42–45)). · When considering the immunization of older adolescents and young adults planning to or currently attending post-secondary education, it is important to consider PT variations in epidemiology and the timing of routine adolescent Men-C-C or Men-C-ACWY vaccination programs. IMD risk in individuals 15–24 years of age may not be limited to their educational status or living situation. · Some Canadian jurisdictions have introduced serogroup B vaccination programs for population groups that are believed to be at higher risk of disease due to increased exposure. However, while inequity may result from PT differences in vaccine access, the ultimate goal of IMD programs should be the reduction of differences in disease outcomes between populations, which is likely to be impacted significantly by regional epidemiology as well as the timing and composition of PT adolescent immunization programs. · Permissive recommendations may lead to potential inequities relative to access and the ability of high-risk individuals to purchase the recommended vaccine(s).
Feasibility	· There are currently six meningococcal vaccines that are purchased by PTs through the national vaccine bulk procurement program and to date there have been no reported shortages. · Given its broader age group indication (2 months–25 years), 4CMenB has been to date the serogroup B vaccine product of choice compared to MenB-FHbp (10–25 years). · University/college aged students may move to attend post-secondary education in a PT with different IMD epidemiology than where they are from. · Program cost and complexities associated with new program implementation should be weighed against the challenges, costs, and limitations of outbreak mitigation and contact tracing in the absence of programs.
Acceptability	· The level of individual and population group risk is influenced by local epidemiology and the timing and type of adolescent PT programs. · Acceptability is likely to be increased as a result of higher awareness of risk among populations that are considered to be at higher risk of IMD. · Vaccine uptake among adolescents and young adults living in shared accommodation settings is likely to be high given the media attention that was focused on previous outbreaks in post-secondary educational settings.
Others	· Canada has aligned with the World Health Organization Global call to action to defeat meningitis by 2030 and provides continued immunization against vaccine preventable serogroups ((46,47)).

Abbreviations: IMD, invasive meningococcal disease; MenB-FHbp, bivalent factor H binding protein meningococcal serogroup B; Men-C-ACWY, meningococcal quadrivalent ACYW conjugate vaccine; Men-C-C, monovalent conjugate meningococcal C vaccine; NACI, National Advisory Committee on Immunization; *Neisseria gonorrhoeae*, *N. gonorrhoeae*; *Neisseria meningitidis*, *N. meningitidis*; PT, provinces and territories; 4CMenB, serogroup B meningococcal vaccines

## Conclusion

In Canada, the age groups with the highest incidence of IMD include children younger than five years of age, followed by people 15–24 years of age. Determining particular risk factors (e.g. those associated with a particular activity or setting) beyond age and underlying medical conditions is challenging due to the limitations of currently collected data. Given that IMD epidemiology varies across the country, jurisdictions with higher incidence in specific population groups may therefore consider introducing targeted programs (e.g. offering a serogroup-appropriate meningococcal vaccine to an age group with a higher incidence of IMD), which may also include populations that are believed to be at higher risk of exposure (e.g. students residing in congregate settings or children and adolescents living in regions with circulating hypervirulent clones). When planning targeted programs, consideration should be given to the specific regional circulating strains and epidemiology.

Due to the PT differences in circulating strains and epidemiology, NACI concluded that recommending a single pan-Canadian program targeting additional population groups at high risk of exposure would be challenging and that regional programs may be better suited to address the currently circulating serogroups and prevent IMD in population groups considered to be at high risk of exposure.

While much is known about IMD, further studies are needed to better understand the contemporary risk factors of IMD in high-incidence population groups (including adolescents and young adults) in Canada, including activities and settings associated with bacterial acquisition, carriage and transmission. In addition, further research is needed with regards to estimating the true cost of IMD and meningococcal infections in Canada, including those associated with the absence of immunization programs (e.g. costs associated with contact tracing, school disruptions, outbreak management, etc.). Robust surveillance systems with enhanced data collection are required for the continuous monitoring of vaccine-preventable diseases, program evaluation and timely adjustment of recommendations that are focused on equity.
